# Secrets of DNA-PKcs beyond DNA repair

**DOI:** 10.1038/s41698-024-00655-1

**Published:** 2024-07-23

**Authors:** Sydney Camfield, Sayan Chakraborty, Shailendra Kumar Dhar Dwivedi, Pijush Kanti Pramanik, Priyabrata Mukherjee, Resham Bhattacharya

**Affiliations:** 1 Department of Pathology, University of Oklahoma Health Sciences Center, Oklahoma City, OK USA; 2 Department of Obstetrics and Gynecology, University of Oklahoma Health Sciences Center, Oklahoma City, OK USA; 3 Peggy and Charles Stephenson Cancer Center, University of Oklahoma Health Sciences Center, Oklahoma City, OK USA

**Keywords:** Cancer therapy, Cell signalling, Chemotherapy

## Abstract

The canonical role of the DNA-dependent protein kinase catalytic subunit (DNA-PKcs) in repairing DNA double-strand breaks combined with its reported dysregulation in several malignancies has driven the development of DNA-PKcs inhibitors as therapeutics. However, until recently the relationship between DNA-PKcs and tumorigenesis has been primarily investigated with regard to its role in non-homologous end joining (NHEJ) repair. Emerging research has uncovered non-canonical DNA-PKcs functions involved with transcriptional regulation, telomere maintenance, metabolic regulation, and immune signaling all of which may also impinge on tumorigenesis. This review mainly discusses these non-canonical roles of DNA-PKcs in cellular biology and their potential contribution to tumorigenesis, as well as evaluating the implications of targeting DNA-PKcs for cancer therapy.

## Introduction

As cells grow and divide they are exposed to extrinsic and intrinsic stressors which result in the accumulation of DNA damage. If left unrepaired, the ensuing DNA breaks threaten genomic integrity. Cells thus have robust mechanisms for alleviating DNA damage that are collectively known as the DNA damage response (DDR), and include cellular checkpoints and DNA repair. Non-homologous end joining (NHEJ) is the most common DNA repair machinery and employs several DDR proteins; a prominent component of NHEJ is the DNA-dependent protein kinase catalytic subunit (DNA-PKcs).

In 1985, Walker et al. first demonstrated that the addition of double-stranded DNA (dsDNA) to multiple cell extracts, mimicked DNA double-stranded breaks (DSB) and triggered the phosphorylation of several proteins^1^. Purification and identification of the DSB-activated proteins revealed a DNA-activated protein kinase, which we know today as DNA-PKcs^2^. Since then, DNA-PKcs and its NHEJ partners have been extensively studied in both DDR and variable, diversity, and joining (V(D)J) recombination. However, the functions and targets of DNA-PKcs beyond NHEJ have not been comprehensively elucidated.

Over the last decade, studies regarding the novel functions of DNA-PKcs have increased significantly. These studies have described not only additional nuclear roles for DNA-PKcs but have also uncovered cytosolic functions. The centrality of DNA-PKcs to DNA repair and various other cellular pathways raises the question of how DNA-PKcs dysregulation contributes to tumorigenesis. Additionally, the endogenous regulation of DNA-PKcs is not well understood, so the basis of the DNA-PKcs dysregulation that occurs in many diseases, including cancer, remains elusive.

In this review, we discuss the emerging novel functions of DNA-PKcs and the consequences of dysregulation that contribute to tumorigenesis, as well as highlight the various DNA-PKcs inhibitors that have been developed and tested in clinical trials thus far.

## Structure and biochemistry

### Domains of DNA-PKcs

**Fig. 1:**
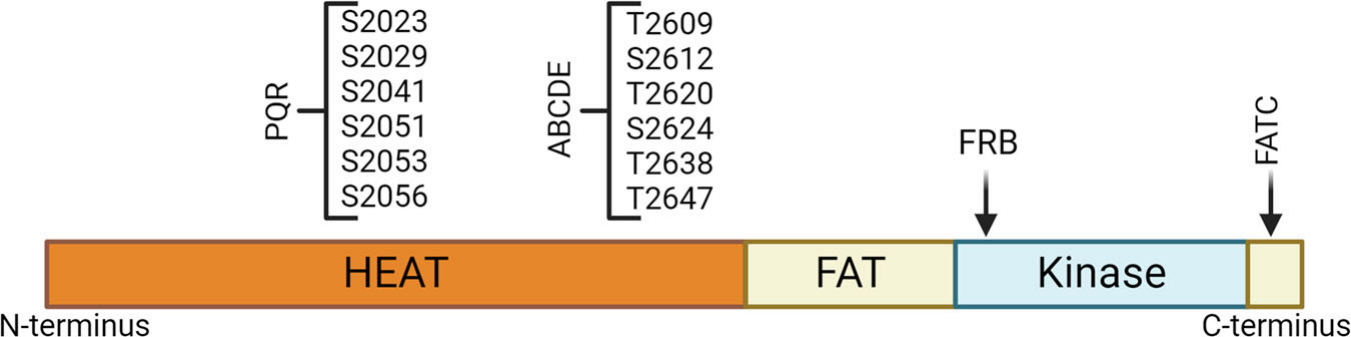
The structural domains of DNA-PKcs—HEAT, FAT, FRB, kinase, and FATC—and prominent phosphorylation clusters. HEAT repeats facilitate binding with DNA and are potentially involved in protein-protein interactions. The FAT and FATC domains mediate the activity of the kinase domain and act as a sensor during the activation of DNA-PKcs. The kinase region is the catalytic region that phosphorylates target ser/thr residues. Within the kinase domain, FRB is speculated to be a “gatekeeper” which further regulates the activity of the protein. Prominent phosphorylation clusters, PQR and ABCDE, are important for the activation and function of DNA-PKcs. Figure created with BioRender.com.

DNA-PKcs is a large serine/threonine (Ser/Thr) kinase consisting of more than 4000 amino acid residues^3^. Sequencing and biochemical assays revealed DNA-PKcs to be a member of the phosphoinositide 3-kinase (PI3K)-related kinase (PIKK) family given its structural similarity to other members of the PI3K family^4,5^. Other PIKK proteins include the mammalian target of rapamycin (mTOR), ataxia-telangiectasia mutated (ATM), and ataxia telangiectasia and Rad3-related protein (ATR) with DNA-PKcs being the largest of all the family members. Despite the significant size variations, all PIKK proteins have similar conserved domains, specifically the HEAT (Huntingtin, Elongation Factor 3, PP2A, and TOR1) repeats, FAT/FATC (FRAP, ATM, TRAPP/C-terminus), FKBP-rapamycin-binding (FRB), and kinase domains (Fig. 1)^6^.

Obtaining a high-resolution structure of all the domains is crucial not only for comprehending the regulation of DNA-PKcs activity but also for identifying novel drug-binding sites and developing structure-based inhibitors. Recently, through high-resolution cryogenic electron microscopy (Cryo-EM), two conformational states of DNA-PKcs have been revealed: intermediate, and active^7^. These different conformational states are a result of the N-terminus arm moving in a hinge-like manner towards the FAT domain^8^. These changes are influenced by the binding of DNA-PKcs to DNA leading to modifications its activity.

The highly conserved, catalytic, kinase domain confers activity to DNA-PKcs. It is recognized that DNA-PK preferentially phosphorylates serines or threonines, followed by glutamines (SQ/TQ motif) on proteins. However, the SQ/TQ motifs are considered the consensus sequences for ATM and ATR, as well as for DNA-PKcs^9^. Using purified proteins in vitro, several substrates of DNA-PKcs have been identified^10^ with many being involved in DDR and cell cycle progression, such as the MET receptor tyrosine kinase and histone 2A family member X (H2AX)^11,12^. Although, it must be noted that more recent targeted phosphoproteomic studies have examined the redundancy between the PIKK family proteins, highlighting the compensatory effects and intricate relationships between these kinases^13^. Preceding the kinase domain is FRB, a four-helix domain that moves outward as a DNA-PKcs binds to Ku, another DDR protein, suggesting that it may function as a “gatekeeper” of kinase actvity^14^. In mTOR, FRB acts as a controller of its kinase activity by blocking access to the kinase domain, further validating the potential “gatekeeper” role of FRB in DNA-PKcs^14^. While FRB’s structure has been elucidated, there is still a need for better functional characterization. If FRB truly controls DNA-PKcs kinase activity, it may be a suitable target for structural-based inhibitors.

While the kinase domain confers activity to the protein, the other domains are just as important to the overall functioning and activation of DNA-PKcs. Protein crystallization and Cryo-EM conformation allowed visualization of the HEAT repeats, identifying a hollow ring structure that facilitates binding of DNA^15,16^. This domain comprises ~65 α-helical HEAT repeats split into an N-terminal (N-HEAT) domain and a middle (M-HEAT) domain. Furthermore, the repeats twist and stretch to produce an active-state structure with kinase abilities^5^. Questions remain as to what additional functional attributes these HEAT repeats may confer to DNA-PKcs. Based on the function of these repeats in other proteins, it can be speculated they may serve as temporary, protein-protein interaction sites.

The final regions are the FAT and FATC domains which flank the kinase domain. The FATC domain possesses important sites that mediate the activity of DNA-PKcs. In the presence of DNA damage, tat-interactive protein 60 kDa (TIP60) activates DNA-PKcs through interaction with the FATC domain^17^. It is speculated this activation occurs through acetylation, the same mode of activation by which TIP60 acts on ATM^18^. Additionally, ATM phosphorylates the FATC domain of DNA-PKcs at threonine-4102 (T4102) in response to DSBs which promotes induction of NHEJ^19^. While functional experimentation on the FAT domain remains scarce in comparison to FATC, Cryo-EM imaging suggests the FAT and FATC domains work together as a sensor to induce conformational changes that alter kinase activity^20^.

Further examination of the various domains and how they work to confer activity or stability to DNA-PKcs is essential in discovering new targets and developing effective inhibitors. Future research on the HEAT repeats and protein-protein interactions involving the HEAT domain is essential. Similarly, examining the FATC domain to uncover additional DNA-PKcs binding partners may provide insight into the stability of DNA-PKcs.

### Phosphorylation clusters in DNA-PKcs

While DNA-PKcs has several phosphorylation sites, the most prominent clusters, specifically termed ABCDE and PQR, both reside in the HEAT domain and contain residues that are important to the function of the protein. Autophosphorylation of ABCDE is critical for efficient NHEJ repair^21,22^. After DNA-PKcs binds to DSBs, it is speculated that the autophosphorylation of residues within ABCDE induces conformational changes which allow dissociation of DNA-PKcs from the DNA and subsequent induction of NHEJ. Additionally, when DNA-PKcs is activated by hairpin DNA, there is a robust autophosphorylation of the ABCDE cluster and subsequent activation of Artemis which cleaves the hairpin to allow for repair^23^. Interestingly, alanine substitutions in the ABCDE cluster slowed DSB repair, significantly delayed resolution of γH2AX foci, and increased sensitivity to ionizing radiation thus validating its critical involvement in NHEJ^24,25^. The most reported residue within the ABCDE cluster is T2609, a marker for IR-damage^26^. While some studies have concluded that phosphorylation within the ABCDE cluster is primarily DNA-PK-dependent autophosphorylation^27^, others report that ATM can also phosphorylate these sites^28^. Additional sites that reside in the ABCDE cluster are serine-2612 (S2612), T2620, S2624, T2638, and T2647 (Fig. 1), though less is known about their contribution to the overall function of DNA-PKcs, necessitating further studies for a complete characterization of this cluster.

The second phosphorylation cluster, PQR, contains residues S2023, S2029, S2041, S2051, S2053, and S2056 (Fig. 1). The most studied site within this region is S2056 due to its autophosphorylation which induces NHEJ and repair^29,30^. Current antibodies utilize phospho-T2609 (p-T2609) or p-S2056 as sites to confirm activation of DNA-PKcs post damage. However, the remaining residues within the ABCDE and PQR clusters cannot be disregarded. Some important unaddressed questions remain; how does phosphorylation of other residues within the clusters alter DNA-PKcs function and do they have roles outside of DNA repair? Research in this direction will further characterize the activity of DNA-PKcs in DNA repair and may uncover additional functions of this classical repair protein.

## Endogenous regulators of DNA-PKcs levels

DNA-PKcs is an abundant, mammalian enzyme with differential expression in human tissues. Maintaining proper levels of DNA-PKcs appears to be critical for cellular survival and genomic stability given that dysregulation is observed in and known to contribute to, various pathological conditions, including Alzheimer’s disease, cancer, and severe combined immunodeficiency (SCID)^31–34^. In cultured human cells, DNA-PKcs was reported to be abundantly present across all cell lines^35^. However, in tissues, while DNA-PKcs showed little variation at the mRNA level there was differential expression of protein, suggesting regulation at the post-transcriptional level^36^. To date, the regulation of DNA-PKcs has primarily been explored at the protein level, and such studies show the regulation of DNA-PKcs through interactions with caspase 3^37^, ubiquitin-proteasomal pathway (UPP) proteins^38–40^, and the Tel2, Tti1, and Tti2 complex^41^.

**Fig. 2:**
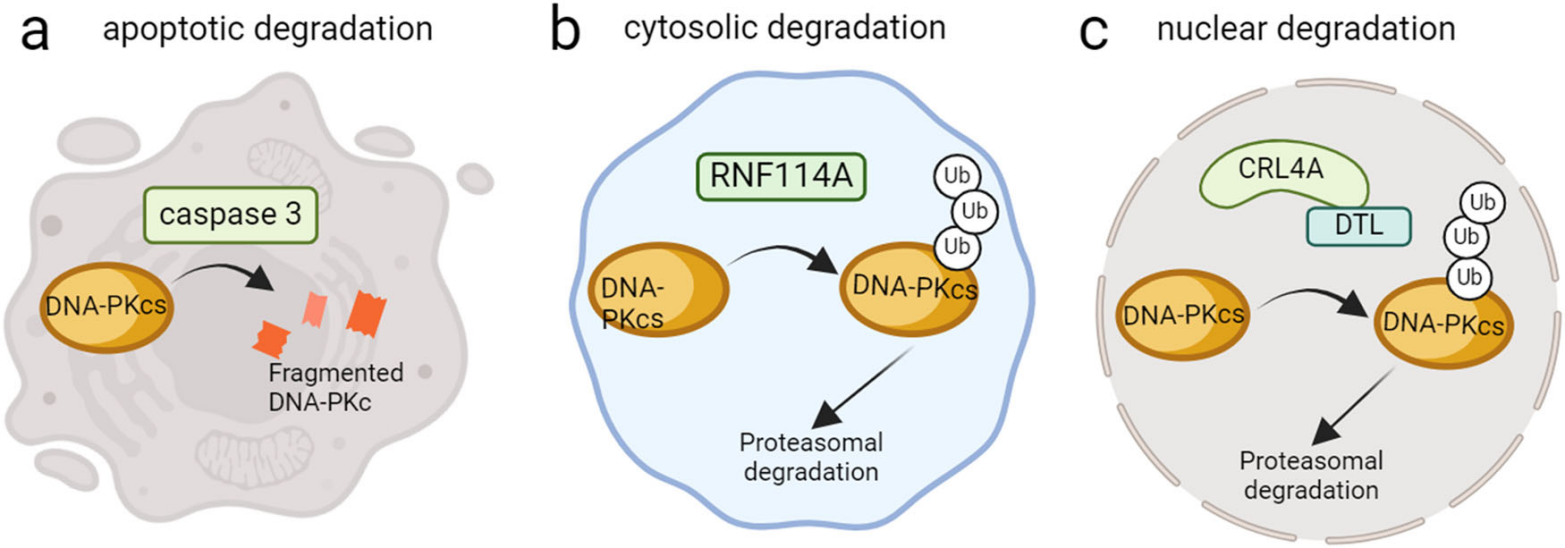
Degradation pathways of DNA-PKcs. **a** Under apoptotic conditions, caspase 3 cleaves DNA-PKcs, promoting efficient apoptosis. **b** The E3 ligase RNF114A targets and ubiquitinates DNA-PKcs to promote cytosolic degradation. **c** Interactions with the E3 ligase, CRL4A, and specific substrate receptor, DTL, are responsible for the nuclear ubiquitination and subsequent degradation of DNA-PKcs. Figures created with BioRender.com.

The first regulatory mechanism observed was the ICE-like protease, CPP32 (caspase 3), an essential apoptotic protein (Fig. 2a)^37^. During etoposide-induced apoptosis, caspase 3 cleaved and fragmented DNA-PKcs. This cleavage is speculated to lead to more efficient apoptosis, which we further discuss in “Roles of DNA-PKcs in cancer biology”.

Further studies have explored UPP-mediated degradation of DNA-PKcs by E3 ligases, i.e., ring finger protein 144A (RNF144A) and cullin ring ligase 4A (CRL4A^DTL^). RNF144A shows p53-dependent accumulation after adriamycin treatment, which also increases cytosolic DNA-PKcs^38^. In the cytosol, RNF114A targets and ubiquitinates cytosolic DNA-PKcs for degradation which is speculated to then down-regulate DNA-PKcs pro-survival functions and thus promote apoptosis (Fig. 2b). Similarly, in the presence of DSBs, CRL4A^DTL^ is recruited to the nucleus where it ubiquitinates DNA-PKcs for degradation, suppresses NHEJ, and increases malignant transformation (Fig. 2c), once again suggesting that DNA-PKcs is degraded after DNA damage to produce more efficient apoptosis^39^. Interestingly, valosin-containing protein (VCP), a chaperone protein that guides ubiquitinated proteins to the proteasome, interacts with ubiquitinated DNA-PKcs and directs it to the proteasome for degradation^40^. VCP knockdown results in the accumulation of DNA-PKcs and promotes DNA repair in glioblastoma cells. Further investigation into UPP-mediated regulation of DNA-PKcs is needed to fully understand the cellular context that necessitates degradation.

Regulation of DNA-PKcs is also achieved via protein stabilization. The Tel2, Tti1, Tti2 (TTT) co-chaperone family is shown the stabilize DNA-PKcs through interactions with inositol hexakisphosphate kinase 2 and casein kinase 2 (CK2)^41^. These interactions allow binding and stabilization of DNA-PKcs to the TTT complex, which then allows activation of p53 and promotion of apoptosis in murine B cells. This suggests that the TTT complex may sequester DNA-PKcs from carrying out its DNA repair functions, leading to p53-dependent apoptosis.

While some aspects of DNA-PKcs regulation have been elucidated, further studies are needed. The downregulation and degradation of DNA-PKcs after DNA damage suggest its pro-survival functions. It remains possible that DNA-PKcs degradation occurs in response to excessive DNA damage or cellular stressors to transition from pro-survival to pro-apoptotic functions. Future research should focus on when and how the degradation mechanisms are employed, and explore new regulatory mechanisms at both transcriptional and translational levels, potentially offering insights for more effective DNA-PKcs-targeting therapeutics.

## Diverse cellular functions of DNA-PKcs

In the following section, the emphasis will be on discussing the emerging non-canonical functions of DNA-PKcs and their implications in cellular and cancer biology with a brief discussion of the canonical roles.

### Canonical roles of DNA-PKcs

The classical roles of DNA-PKcs are in NHEJ, the dominant DNA repair pathway, and in V(D)J recombination, an essential step in lymphocyte development. While the function of DNA-PKcs in both cases is to repair DSBs, NHEJ and V(D)J recombination serve entirely different purposes. NHEJ is the most widely utilized repair pathway due to its ability to repair IR-induced DNA DSBs during any phase of the cell cycle. Recent reports indicate that the process of NHEJ starts with the deacetylation of DNA-PKcs by sirtuin 2 (SIRT2) in response to DNA damage^42^. This facilitates the localization of unphosphorylated DNA-PKcs to the DSBs and subsequent interaction with Ku proteins^43^. The complex that forms between DNA, Ku70/80, and DNA-PKcs creates a repair complex called DNA-dependent protein kinase (DNA-PK). These DNA-PK complexes have both protective and processing abilities and can distinguish between different types of breaks and employ the appropriate mechanism^44^. DNA-PKcs recognizes blunt ends, overhangs, or hairpins. Hairpins and overhangs induce the DNA-PK processing complex. For hairpin repair, DNA-PKcs phosphorylation via ATM or trans-autophosphorylation is required with recruitment and phosphorylation of the nuclease Artemis facilitating end opening^45^. In contrast, blunt ends are protected by DNA-PKcs, and subsequent autophosphorylation occurs at a slower rate^46^. Once a processing or protective complex is formed, Artemis uses its endonuclease activity to process the DNA, and X-ray repair cross-complementing protein 4 (XRCC4), XRCC4-like factor (XLF) and DNA ligase IV (Lig4) ligate the strands to complete repair. While NHEJ repair is well-characterized, the precise kinase role and fate of DNA-PKcs after autophosphorylation remains unknown. Gaining insight into the autophosphorylation and chromatin-association consequences of DNA-PKcs after autophosphorylation will help complete the picture of NHEJ.

The second classical role of DNA-PKcs is in V(D)J recombination, where NHEJ machinery is integral in creating diverse antigen receptors in immature T and B cells through the rearrangement and repair of DNA segments^47^. In SCID mice, mutation of the *Prkdc* gene that encodes DNA-PKcs causes defective V(D)J recombination and halts B and T lymphocyte development^48,49^. V(D)J involves lymphocyte-specific endonucleases, known as recombination activating genes (RAG1 and RAG2), which create DSBs with hairpin-like ends. DNA-PKcs along with Artemis work to open the hairpins and Ku70/80, XRCC4, and Lig4 repair the break to complete recombination and formation of new antigen receptor^50,51^.

These classical roles of DNA-PKcs have long been studied and the mechanisms are now well characterized. Future efforts should focus on how diseases and pathological conditions dysregulate DNA-PKcs and subsequently affect NHEJ and V(D)J recombination efficiency and function. Additionally, illuminating the roles of DNA-PKcs outside of DNA repair will allow for a better characterization of the kinase.

### Non-canonical roles of DNA-PKcs in repair and telomere maintenance

It is worth noting the role of DNA-PKcs in homologous recombination (HR) and single-stranded break (SSB) repair. While inhibiting DNA-PKcs and thereby NHEJ may facilitate repair via HR, the emerging picture is complex^52–54^. Certain phosphorylations of DNA-PKcs can promote HR and inhibit NHEJ suggesting DNA-PKcs status impacts which repair pathway is utilized^54^. Additionally, DNA-PKcs may work to repair SSBs by forming repairosomes and base excision repair complexes with XRCC1, Polβ, and APE^55^. However, the involvement of DNA-PKcs in these repair mechanisms is relatively underexplored and requires further validation.

A more definitive role for DNA-PKcs along with Ku has been described in telomere maintenance where Ku86 and DNA-PKcs localize at telomeres^56^. In DNA-PKcs deficient mice, both shortened telomeres and early-onset aging are observed when compared to their wild-type littermates^57^. Further mechanistic studies reveal DNA-PKcs is recruited to the middle of the telomere where it associates with telomere-specific proteins, TRF1 and TRF2, part of the shelterin complex, to mediate telomere maintenance^58^. Additionally, the human telomerase RNA component (hTR) activates DNA-PKcs which allows for subsequent phosphorylation of heterogeneous nuclear ribonucleoprotein A1 (hnRNP A1) and telomere capping^59^. For an elaborate review on DNA-PKc and its role in telomere maintenance, please refer to ref. ^60^.

### DNA-PKcs role as a transcriptional regulator

Alongside roles in DNA repair, DNA-PKcs also function within the nucleus to regulate transcription. In 1990 it was discovered that DNA-PKcs binds the GC-rich regions of cellular promoters. The authors reported that Sp1, a mammalian transcription factor, forms a transcriptional complex with DNA-PKcs, becomes phosphorylated, and induces transcription^61^. This observation led to additional studies examining DNA-PKcs’ influence on gene expression through interactions with transcription factors. A study using Chinese hamster ovarian cells lacking Ku70/Ku80 or DNA-PKcs demonstrated a substantial decrease in transcription across multiple promoters when compared to control cells^62^. This underscores the essential and extensive role played by DNA-PKcs in various transcriptional processes. However, the study fails to mechanistically demonstrate the role of DNA-PKcs in the alterations of these pathways. Therefore, additional studies are needed to elucidate the exact interacting partners of DNA-PKcs.

Several studies have directly demonstrated DNA-PKcs’ influence on transcriptional regulation. DNA-PKcs affects general transcription through the phosphorylation of tripartite motif-containing 28 (TRIM28), a modification vital for the activity of RNA polymerase II^63^, TATA-binding protein (TBP), transcription factor IIB (TFIIB)^64^, and c-MYC^65^ ultimately enhancing basal transcription. Using chromatin immunoprecipitation, Ju et. al., demonstrated that following ligand stimulation DNA-PKcs and Ku70/86 proteins co-purified with DNA topoisomerase IIβ at transient, site-specific DSBs on the nuclear receptor promoters. Subsequently, poly(ADP-ribose) polymerase 1 (PARP1) enzymatic activity was induced and was required for a nucleosome-specific histone H1-high-mobility group B exchange event, ultimately leading to transcriptional activation^66^. They concluded that the TopoIIβ-dependent DNA break represented a widespread strategy for regulated gene transcription, especially for nuclear receptors.

Cell survival is influenced by DNA-PKcs via direct phosphorylation and activation of the POU domains of octamer-binding transcription factors 1 and 2 (Oct-1 and Oct-2) facilitated by Ku which promotes survival following IR-induced damage^67^. Under hypoxic conditions, DNA-PKcs phosphorylates TRIM28 which forms a hetero-trimer with hypoxia-inducible factor-1α (HIF-1α) and binds to the hypoxia-responsive element of HIF target genes to promote survival^68^. Additionally, DNA-PKcs phosphorylates transcription factors, such as early growth response 1 (EGR1) and nuclear factor of activated T-cells (NFAT), influencing the production of interleukin-2 in activated human Jurkat T cells^69,70^.

Metabolic pathways are altered by DNA-PKcs through targeting of the transcription factor TAF7 which leads to the upregulation of RAPTOR and subsequent activation of mTORC1^71^. Lastly, the DNA-PK complex interacts with the transcription factor upstream stimulatory factor-1 (USF-1) where DNA-PKc phosphorylates USF-1 which in turn regulates various genes involved in lipid and carbohydrate metabolism^72^.

**Fig. 3:**
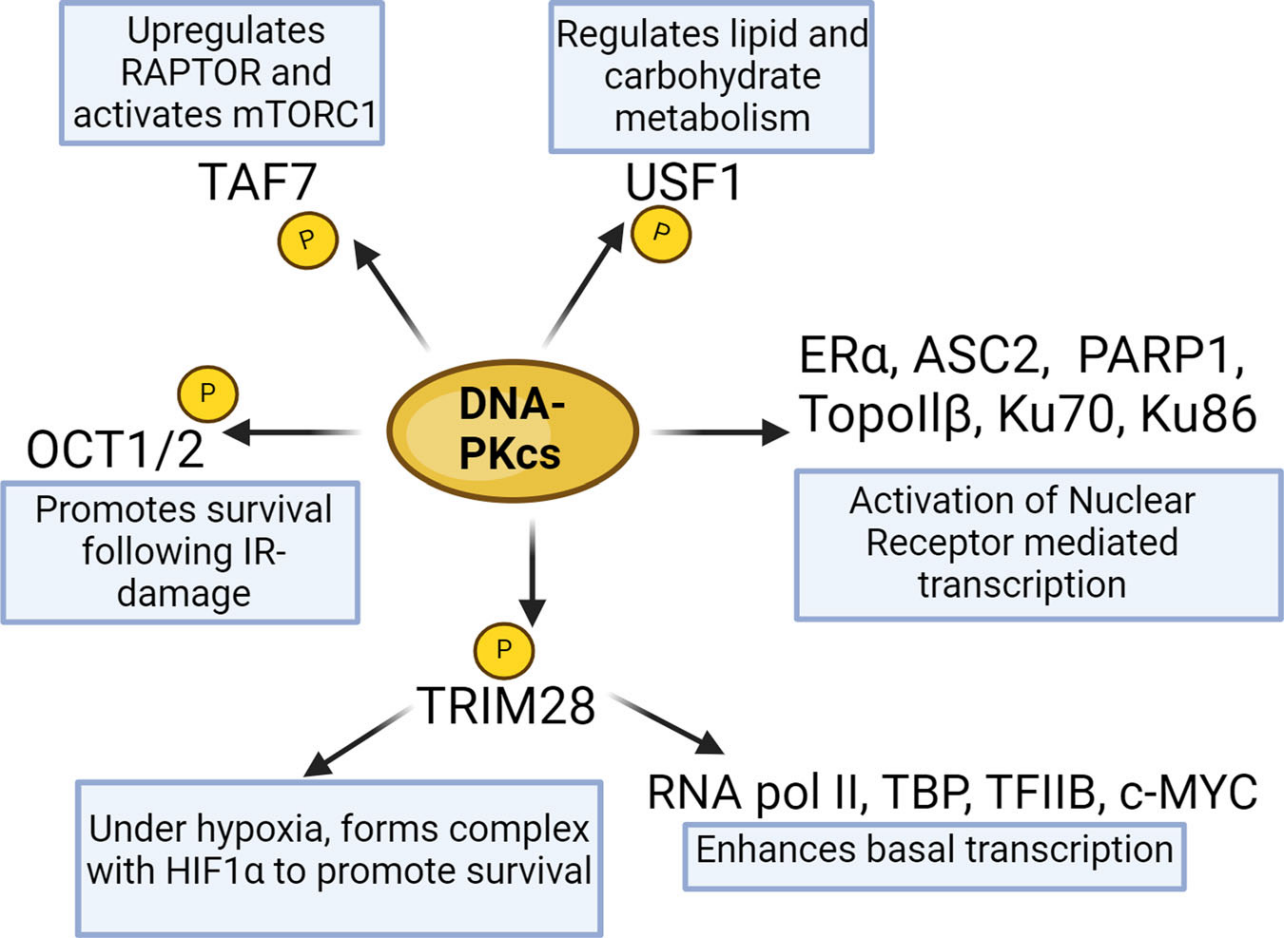
DNA-PKcs acts as a transcriptional regulator. Several transcription factors coordinate with DNA-PKcs to regulate a variety of cellular signaling. Figure created with Biorender.com.

In summary, DNA-PKcs, initially known for its DNA repair functions, has emerged as a multifaceted player in transcriptional regulation (Fig. 3). Unveiling the complexity of DNA-PKcs in transcriptional regulation is essential for a comprehensive understanding of its physiologic and pathologic roles.

### DNA-PKcs in metabolic regulation

As elaborated above DNA-PKcs has prominent nuclear functions. However, DNA-PKcs is also found in the cytoplasm, begging the question of possible roles in this compartment^73^.

**Fig. 4:**
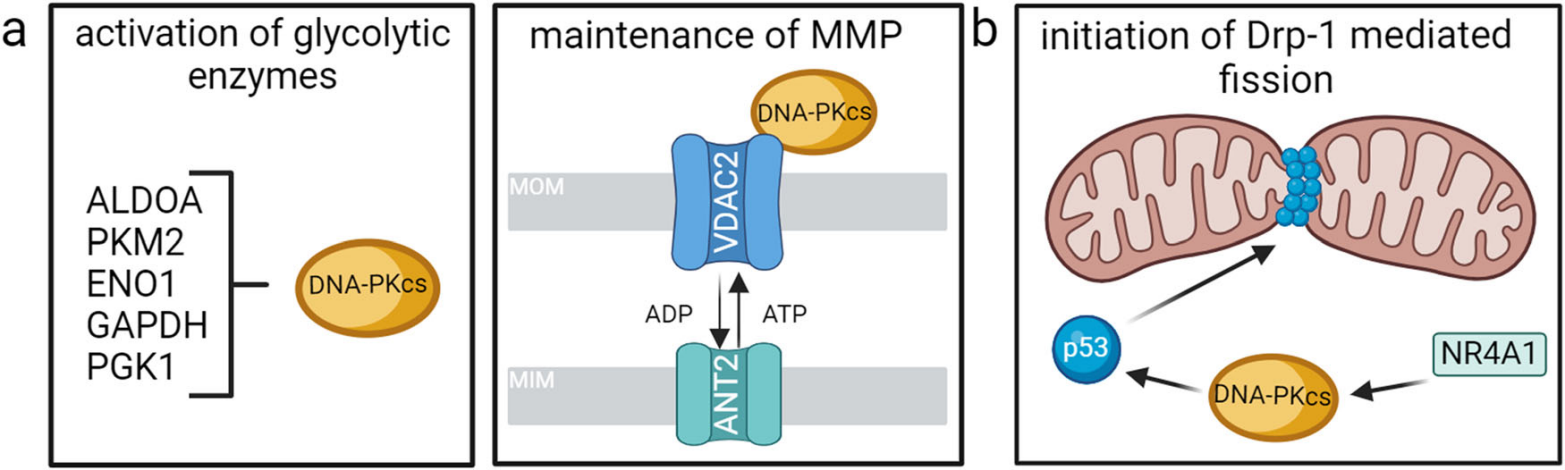
DNA-PKcs in metabolic pathways. **a** DNA-PKcs influences cellular respiration through the activation of glycolytic enzymes and the formation of a complex with VDAC2/ANT2 which maintains the mitochondrial matrix membrane potential. **b** NR4A1-activated DNA-PKcs activates p53 and induces Drp-1-mediated mitochondrial fission. Figures created with BioRender.com.

To date, two studies have shown a role for DNA-PKcs in cellular respiration (Fig. 4a). The first showed that DNA-PKcs phosphorylates and activates the key enzymes aldolase, fructose-bisphosphate A (ALDOA) and pyruvate kinase muscle isozyme M2 (PKM2), and also interacts with the glycolytic enzymes enolase 1, glyceraldehyde 3-phosphate dehydrogenase (GAPDH), and phosphoglycerate kinase 1 (PGK1)^73^. The second revealed that DNA-PKcs forms a complex with adenine nucleotide translocase 2 (ANT2) and voltage-dependent anion channel 2 (VDAC2), which regulates the exchange of adenosine triphosphate (ATP) and adenosine diphosphate (ADP) across the membrane maintaining efficient mitochondrial membrane polarization (MMP) and driving oxidative phosphorylation^74^. Under oxidative stress, ATM phosphorylates DNA-PKcs at T2609 which destabilizes the complex and disrupts MMP. Taken together, these findings demonstrate that DNA-PKcs positively regulate cellular respiration.

DNA-PKcs also play a role in the maintenance of mitochondrial homeostasis. In alcohol-related liver disease (ARLD), DNA-PKcs suppresses FUN14 domain containing 1 (FUNDC1)-required mitophagy and elevates Drp1-mediated mitochondrial fission through the nuclear receptor A41 (NR4A1)/DNA-PKcs/p53 axis which aggravates ARLD (Fig. 4b)^75^. Inhibition of this same pathway mitigates high-fat diet-induced non-alcoholic fatty liver disease by decreasing Drp1-mediated fission, inducing mitophagy, and improving overall mitochondrial function^76^. Similarly, increased DNA-PKcs levels following ischemia-reperfusion injury, negatively impacted mitochondrial structure and function by promoting the degradation of Bax inhibitor-1 (BI-1). Conversely, knockout of DNA-PKcs shows stabilization of BI-1, which results in a reduction of oxidative stress, induced mitophagy, and reduced fission^77^.

Lastly, in response to ionizing radiation or aging stress, DNA-PKcs phosphorylates the prominent cytoplasmic protein HSP90a disrupting its chaperone abilities towards AMP-activated protein kinase (AMPK)^78^. AMPK is crucial for mitochondrial biogenesis and maintaining energy metabolism. Therefore, lowering DNA-PKcs enhances AMPK activity, prevents weight gain, preserves mitochondrial function, and sustains physical fitness in middle-aged mice. Importantly, this effect extends to protecting against the development of type 2 diabetes.

In summary, cytosolic DNA-PKcs affects key metabolic pathways involved in cellular respiration and mitochondrial homeostasis. While glycolysis is positively regulated, DNA-PKcs appear to negatively affect mitochondrial homeostasis and aging, implying that under certain contexts inhibition of DNA-PKcs may be advantageous. However, additional studies are needed to fully appreciate the importance of cytosolic DNA-PKcs.

### Involvement of DNA-PKcs in regulating immune functions

**Fig. 5:**
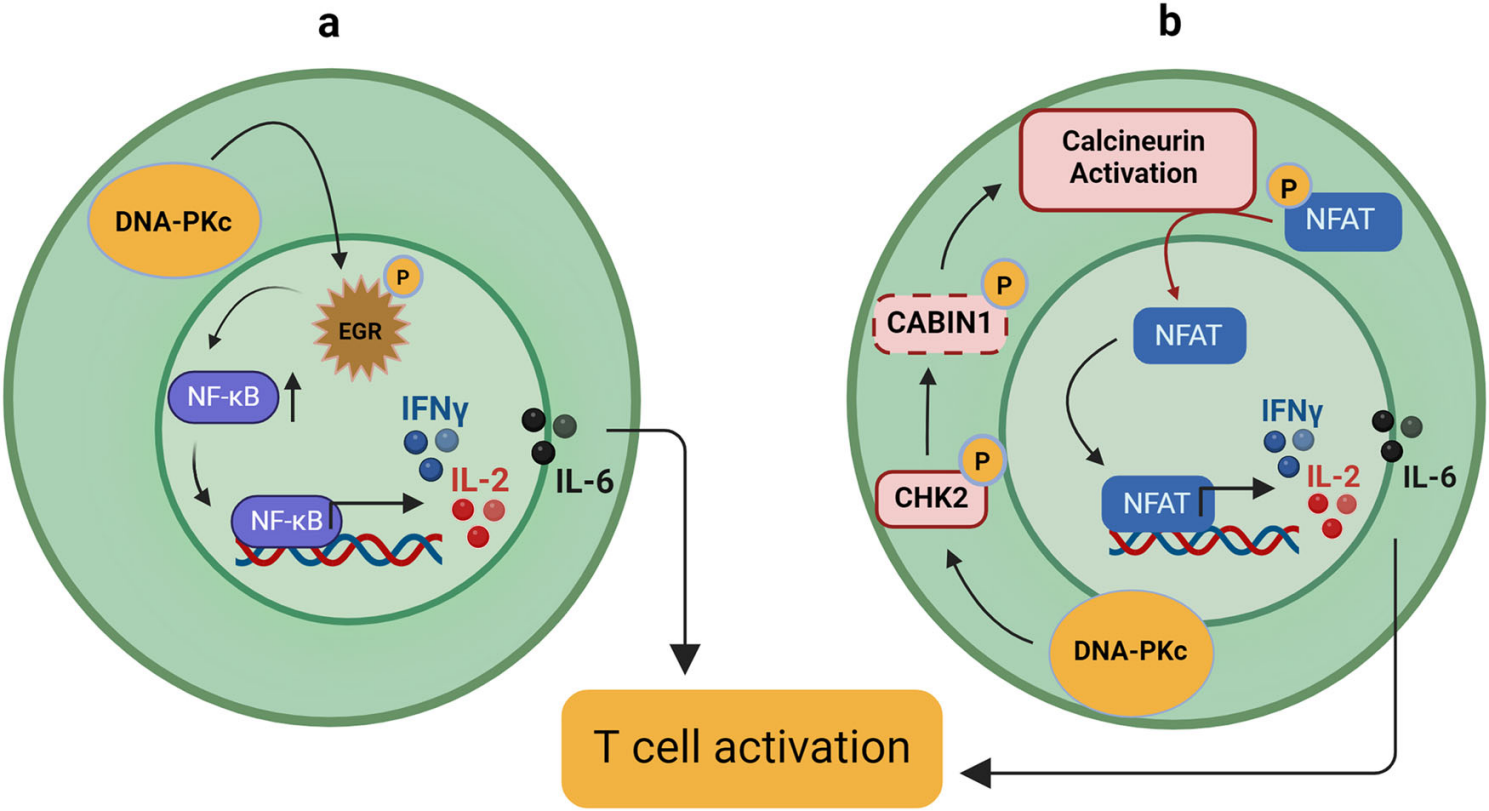
DNA-PKcs orchestrates immune responses through T cell activation. DNA-PKcs can transcriptionally regulate certain key components which determine T cell activation and fate decision. **a** DNA-PKcs induces Egr1 phosphorylation which further activates NFkB to promote various cytokine and co-stimulatory molecule expression. **b** DNA-PKcs promotes NFAT nuclear translocation in a calcineurin dependent manner, which is mediated by DNA-PKcs dependent phosphorylation of CHK2 followed by CABIN1 phosphorylation and degradation leading to the expression of IL-2 and other factors. Figures created with BioRender.com.

Recent studies have revealed roles for DNA-PKcs in the adult immune system beyond its recognized role during development. As previously mentioned, DNA-PKcs have transcriptional functions that can influence immune function. The phosphorylation of Egr1 by DNA-PKcs influences cytokine and costimulatory molecule expression, including expression of IL2, IL6, IFNγ, and NFκB (Fig. 5a)^69^. Inhibiting DNA-PKcs with the small molecule inhibitor NU7441 or with shRNA increased Egr1 degradation, while mutation of serine 301 to alanine reduced Egr1 levels and IL2 transcription in activated T cells. Moreover, DNA-PKcs plays a pivotal role in the calcineurin-mediated translocation of NFAT to the nucleus^70^. Inhibition of DNA-PKcs blocks calcineurin activity, preventing NFAT translocation and IL2 expression (Fig. 5b). These findings emphasize DNAPKc as a vital link between T cell activation and fate.

**Fig. 6:**
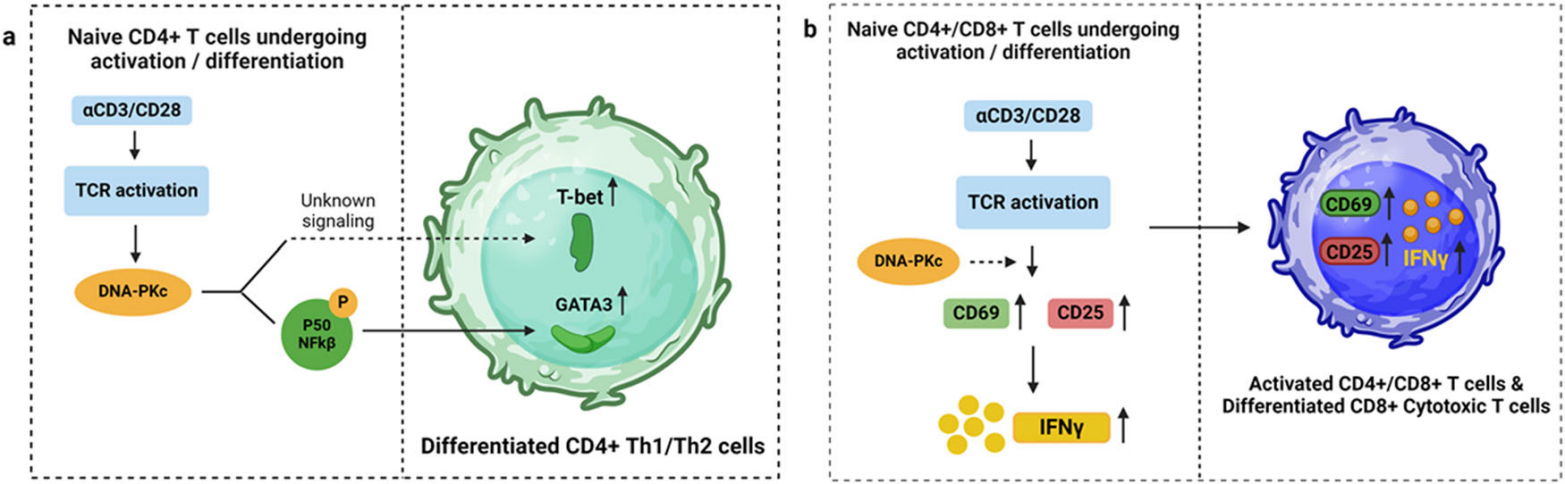
DNA-PKc governing T_H_ and T_C_ differentiation. **a** In naive CD4+ T_H_ cells, upon TCR activation DNA-PKcs stimulates the expression of two key transcription factors T-bet and GATA3 which play pivotal roles in T_H_1 and T_H_2 differentiation respectively. **b** In naive CD4+ T_H_ and CD8+ T_C_ cells, upon TCR activation DNA-PKcs induces expression of CD69 and CD25 along with IFNγ, ultimately promoting activation of T cells and differentiation of CD8+ T_C_ cells into cytotoxic T cells. Figures created with BioRender.com.

In CD4+ T cells, DNA-PKcs governs the expression of both T-bet and Gata3, highlighting its role as a master regulator of Th1 and Th2 differentiation (Fig. 6a)^79,80^. DNA-PKcs is also involved in naive CD8+ T cell activation as well as differentiation into cytotoxic T cells^81^. Using pharmacological and genetic approaches Azevedo-Pouly et. al., demonstrated that inhibiting DNA-PKcs disrupted the activation of CD8+ T cells triggered by anti-CD3/CD28, leading to reduced expression of activation markers CD69 and CD25 with diminished IFNγ production (Fig. 6b)^81^. Additionally, loss of DNA-PKcs activity impaired aerobic glycolysis and the proliferation of OTI-CD8+ T cells when stimulated with the SIINFEKL peptide. This resulted in a decreased capacity for OTI-CD8+ T cells to eliminate MC38.OVA tumor cells and reduced expression of cytotoxic genes, perforin, and granzyme B^81^.

In innate immune cells, DNA-PKcs regulates inflammatory responses. For instance, bone marrow-derived dendritic cells lacking DNA-PKcs show delayed responses to cytosine-phosphorothioate-guanine oligodeoxynucleotides (CpG-ODN), leading to reduced IL-6 and IL-16 production^82^. Similarly, DNA-PKcs-deficient macrophages exhibit impaired inflammatory responses, resulting in reduced IL-10 and IL-18 production in SCID mouse-derived peritoneal and bone marrow macrophages stimulated with thioglycollate^83^. Studies suggest that DNA-PKcs and Ku70/80 both contribute to DNA-PK-mediated immune suppression. DNA-PKcs phosphorylates cGAS, inhibiting its enzymatic activity and reducing IFNB1 and CXCL-10 expression in THP-1 monocytic cells, rendering them more susceptible to viral infection^84^. Additionally, DNA-PKcs in bone marrow-derived macrophages activate AKT (AKR thymoma), promoting its nuclear translocation, which may be essential for AKT-specific targets such as IKK and NF-κB^85^.

Beyond the recognition of self-DNA, DNA-PKcs also detect foreign DNA thus impacting viral infection. Various components of the DNA-PKcs complex can initiate or skew inflammatory responses, leading to type I or type III interferon production in response to non-self dsDNA. For example, KU70 triggers type III interferon responses independently of DNA-PKcs by stimulating IRF1 and IRF7^86^. In contrast, DNA-PKcs itself significantly contributes to antiviral responses, promoting IRF3 phosphorylation after DNA sensing and DNA virus infections^87^. Recently DNA-PKcs has also been shown to promote HIV transcription by interacting with the HIV genome. Although these findings are suggestive of DNA-PKcs’ role in the inflammatory response, more in-depth studies are required to establish the molecular and functional connections of DNA-PKcs complexes with immune responsiveness^88^.

**Fig. 7:**
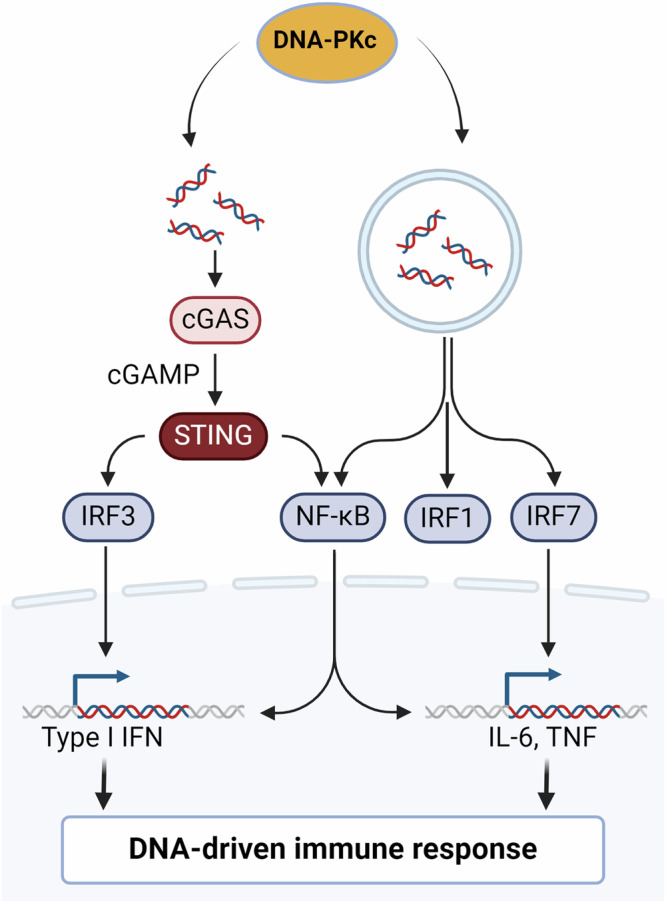
cGAS/STING dependent and independent DNA-driven immune responses are regulated by DNA-PKcs. Self and non-self, DNA-driven immune responses are regulated by DNA-PKcs via IRFs and NFkB in a cGAS/STING dependent or independent manner in several types of cells including epithelial cells, fibroblasts, monocytes, and T cells. Figure created with BioRender.com.

Some DNA viruses have evolved strategies to avoid detection by DNA-PKcs, while others exploit NHEJ for replication, highlighting the intricate relationship between viral life cycles and the DDR. The precise coordination of DNA-PKcs-dependent inflammatory responses and STING activation by IFI16 and cGAS remains unclear. There is an ongoing debate about whether DNA-PKcs requires STING for interferon production, with various scenarios proposed. Some studies suggest that DNA-PKcs is recruited to cytoplasmic dsDNA via KU80, initiating IRF3-dependent inflammatory responses, either dependent or independent of DNA-PKcs catalytic activity (Fig. 7). Additionally, DNA-PKcs can work independently of STING^89^ while inhibiting cGAS enzymatic activity^84^. The interplay between DNA-PKcs and cGAS-STING activation may also vary depending on the cell cycle stage^90,91^. Moreover, DNA-PKcs’ immune signaling appears species-specific, with differences between human and murine cells. In human cells, DNA-PKcs can activate innate immune responses independently of STING, while in murine cells, it may signal through STING **(**Fig. 7**)**^87^. The role of DNA-PKcs in inducing type I interferon responses may also be subject to cell type-specific regulatory mechanisms, similar to the regulation of IFI16 activation, requiring further investigation.

## DNA-PKcs in cancer

### Roles of DNA-PKcs in cancer biology

**Fig. 8:**
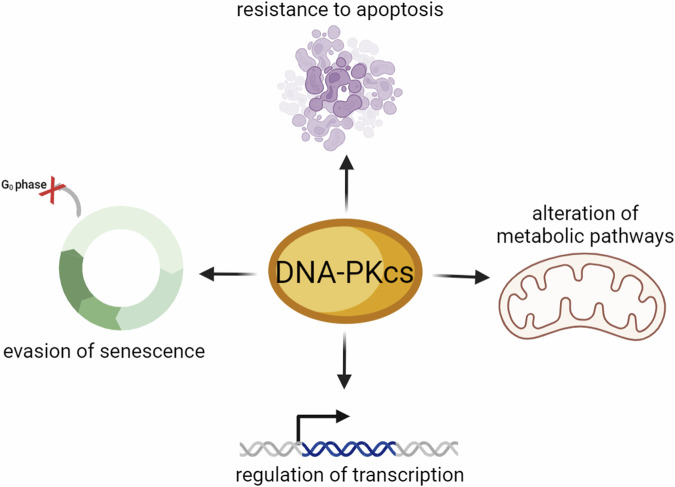
The non-canonical roles of DNA-PKcs which may contribute to tumorigenesis. Non-repair functions of DNA-PKcs include resistance to apoptosis, evasion of senescence, and transcriptional and metabolic regulation. Figure created with BioRender.com.

The impact of DNA-PKcs on tumorigenesis has been extensively studied with regard to its role in genomic stability and continues to be a topic of interest as non-repair functions are being uncovered (Fig. 8).

DNA-PKcs promotes evasion of senescence and resistance to cell death. In DNA-PKcs-inhibited non-small cell lung cancer (NSCLC) cells, irradiation-induced DSBs promote a p53-dependent accelerated senescence determined through senescence-associated beta-galactosidase activity^92^. Similarly, irradiated cancer cells with DNA-PKcs inhibition result in morphological phenotypes and epigenetic changes suggestive of the induction of an irreversible senescent state^93^. Additionally, DNA-PKcs has anti-apoptotic roles through association with AKT. During high reactive oxygen species stress in PC12 cells, DNA-PKcs phosphorylates S473 on AKT thus promoting cell survival^94^. Lastly, through interactions with Ku70 and DNA-PKcs, nuclear Recepteur d’Origine Nantais kinase (RON), also known as macrophage-stimulating protein receptor (MST1R), activates NHEJ and contributes to chemoresistance^95^. These results suggest that in addition to promoting repair, DNA-PKcs is intricately linked with the suppression of cell death and evasion of senescence which together may ultimately contribute to tumorigenesis.

By regulating transcription, DNA-PKcs promotes invasion and metastasis, another hallmark of cancer^96^. In castration-resistant prostate cancer (CRPC), upon stimulation with dihydrotestosterone, DNA-PKcs associates with the androgen receptor (AR) and is recruited to the regulatory loci of AR target genes^97^. Transcriptional networks selectively associated with tumor progression and pro-metastatic signaling are activated resulting in DNA-PKcs-induced tumor cell migration and invasion. Pharmacological inhibition of DNA-PKcs prevents the formation of metastases in vivo. Additionally, irradiation of MCF7 and DLD1 cell lines induces Snail1 phosphorylation at S100 leading to increased protein stability of Snail1, a key epithelial to mesenchymal transition (EMT) transcription factor^98^. Phosphorylated Snail1 reciprocally inhibits the kinase activity of DNA-PKcs, resulting in an inhibition of DNA repair activity. Together this axis promotes the migration of tumor cells and an increase in genomic instability. In breast cancer, DNA-PKcs stabilizes and activates the transcription factor estrogen receptor-α (ERα) which regulates genes involved in cellular growth^99^. Inhibiting DNA-PKcs, either through pharmacological means or by siRNA, decreases ERα activation while increasing its ubiquitination and subsequent degradation. Hence, in several cancers by regulating transcription, DNA-PKcs promotes metastasis.

Lastly, DNA-PKcs influence cancer cell metabolism and cellular energetics. Indirect stabilization of c-MYC via activation of the AKT/GSK3 (glycogen synthase kinase 3) pathway by DNA-PKcs promotes cancer cell growth and metabolism^100^. Reducing DNA-PKcs expression decreases Myc protein levels, suggesting a dual role for DNA-PKcs. As mentioned in “DNA-PKcs in metabolic regulation”, DNA-PKcs also influences glycolysis through the activation of glycolytic enzymes. In CRPC, inhibition of DNA-PKcs slows proliferation despite ectopic expression of the glycolytic enzymes ALDOA and PKM2^73^. Together, these pleiotropic cellular functions of DNA-PKcs suggest it may be a promising therapeutic target but will need to be leveraged in the context of overexpression.

The dysregulation of DNA-PKcs is a common phenomenon in various cancers and correlates with metastasis, invasion, and poor survival outcomes. Overexpression, the most common form of DNA-PKcs dysregulation, is observed in breast cancer^101^, prostate cancer^102^, NSCLC^103^, gastric cancer^34^, ovarian cancer^104^, pancreatic ductal adenocarcinoma (PDAC)^105^, and hepatocarcinoma^106^. One possible explanation for the overexpression of DNA-PKcs in cancer is through the NEAT1/miR-101/DNA-PKcs axis. The micro-RNA, miR-101, targets and binds the 3’ UTR of DNA-PKcs to negatively regulate DNA-PKcs expression. In cancer cell lines, exogenous overexpression of miR-101 decreases DNA-PKcs levels and sensitizes tumor cells to radiation^107^. However, in PDAC, NEAT1, an lncRNA that binds and inactivates miR-101, is upregulated suggesting it may bind miR-101 contributing to DNA-PKcs stabilization and overexpression^108^. While observed less frequently, downregulation of DNA-PKcs in gastric cancer correlates with metastasis and poor survival^109^. In cases of protein overexpression, the inhibition of DNA-PKcs is promising as it would not only reduce the DNA repair abilities of the tumor but may also downregulate transcriptional programs and metabolic pathways that contribute to tumor progression and metastasis. In tumors presenting unchanged levels of DNA-PKcs, inhibition may still be advantageous in combination with chemotherapeutics or radiation, which are genotoxic, necessitating DNA repair. Thus, inhibition of DNA-PKcs has been an active area of research with several inhibitors undergoing clinical trials.

### Current DNA-PKcs inhibitors

To date, various generations of DNA-PKcs inhibitors have been developed. Early-generation inhibitors, such as NU7026 and NU7441, were selective, effective in vitro, and demonstrated proof of function^110^. In vivo, these inhibitors decreased tumor growth when combined with radiation or chemotherapy. However, they could not be translated clinically because of poor pharmacokinetics (PK) and poor oral bioavailability^111,112^. Over the years, several other DNA-PKcs inhibitors have either not progressed to clinical trials or performed unfavorably in trials. Here, we will discuss those inhibitors that have shown promising results in completed trials or are being evaluated in ongoing trials. For a more comprehensive list of DNA-PKcs inhibitors, as well as information on IC_50_ and chemical structures, please refer to ref. ^113^.

As the structural characterization of DNA-PKcs has improved, so have the inhibitors. Many of the later-generation inhibitors have a higher selectivity for DNA-PKcs than for other PIKK/PI3K proteins, as well as a higher potency that makes them translatable (Table 1). The most widely researched, small molecule inhibitor (SMI) of DNA-PKcs is peposertib, also known as M3814, MSC2490484A, or nedisertib. Many in vitro and in vivo studies have demonstrated that peposertib sensitizes cancer cells to radiation and chemotherapy leading to regression of tumors in xenograft mouse models^92,114–117^. These results show peposertib to be both selective and potent and led to the evolution of this inhibitor in clinical trials. Four completed phase I and II clinical trials have demonstrated peposertib to be well-tolerated. The first-in-human, phase 1 clinical trial evaluated peposertib as monotherapy in patients with advanced solid tumors or chronic lymphocytic leukemia (NCT02316197). Out of 31 patients, the best overall response was in 12 patients who presented with stable disease (SD), which lasted 12 weeks or longer in seven patients. Additionally, peposertib was quickly absorbed and primarily produced non-serious adverse effects (AEs), such as gastrointestinal issues, fatigue, and pyrexia^118^. Peposertib was also examined in combination with radiotherapy (RT) or RT plus cisplatin in patients with advanced solid tumors (NCT02516813); the recommended phase II dose of peposertib in combination with RT was 200 mg once daily while with RT and cisplatin it was 50 mg although this latter study arm was terminated as there was insufficient exposure at this low dose^119^. Promising results from these studies prompted further clinical evaluations. Currently, phase I and II trials in CRPC, ovarian cancer, leukemia, neuroendocrine tumors, and glioblastoma are ongoing; examining the effect of peposertib in combination with other therapies including radionuclide therapy, RT, pegylated liposomal doxorubicin (PLD), mitoxantrone, etoposide, and cytarabine (MEC), tuvusertib, temozolomide, and avelumab (Table 1). Interestingly, the PD-L1 immune checkpoint inhibitor (ICI), avelumab, is being evaluated alongside peposertib in patients with advanced solid tumors. Further discussed in the next section, DNA-PKcs knockout cells respond more effectively to ICIs suggesting an advantage in combining DNA-PKcs inhibition with immunotherapies.

**Table 1:** Recently completed and currently ongoing clinical trials targeting DNA-PKcs

Inhibitor	Mode of Inhibition	Additional interventions	Phase	Location	Status/Trial
Peposertib (M3814, MSC2490484A, Nedisertib)	selective SMI	Radium-223 dichloride, Avelumab	I/II	advanced castrate-resistant prostate cancer	Active NCT04071236
		PLD	I/Ib	recurrent low or high grade ovarian cancer	Recruiting NCT04092270
		MEC	I	relapsed or refractory acute myeloid leukemia	Recruiting NCT03983824
		Tuvusertib (M1774)	I	advanced solid tumors	Recruiting NCT05687136
		Lutetium 177 Dotatate	Ib	neuroendocrine tumors	Recruiting NCT04750954
		radiation	I	Advanced head and neck cancer, cannot take cisplatin	Recruiting NCT04533750
		Radiation, temozolomide	I	newly diagnosed MGMT unmethylated glioblastoma or gliosarcoma	Recruiting NCT04555577
		Avelumab, radiation	II	advanced solid tumors	Completed (8-17-2022) NCT03724890
CC-115	dual inhibitor of mTOR and DNA-PK	Monotherapy	II	Glioblastoma	Terminated NCT02977780
		Enzalutamide (Enza)	I	castration-resistant prostate cancer (CRPC)	Completed (1-13-2023) NCT02833883
AZD7648	selective SMI	PLD	I/IIa	advanced solid tumors	Completed (12-07-2022) NCT03907969
XRD-0394	SMI, dual inhibitor of ATM and DNA-PK	Radiation	I	metastatic, locally advanced, or recurrent cancer	Active NCT05002140
AsiDNA	Sequesters DNA-PKcs and other DNA repair proteins	olaparib	Ib/II	recurrent solid tumors	Recruiting NCT05700669
		Niraparib, Olaparib, Rucaparib	I/II	Relapsed Platinum Sensitive Ovarian Cancer	Recruiting NCT04826198
		monotherapy	I	Advanced solid tumors	Completed 2-20-2022 NCT03579628

CC-115, a dual inhibitor of DNA-PKcs and mTOR, sensitized lung cancer cell lines to etoposide, as well as suppressing NSCLC xenograft growth in vivo^120,121^. The first-in-human trial, completed in 2021, examined CC-115 in patients with advanced solid tumors (NCT01353625). CC-115 was well-tolerated with toxicities comparable to those of other mTOR inhibitors. While outcomes varied, SD was achieved in 53%, 22%, 21%, and 64% of patients with head and neck squamous cell carcinoma, Ewing sarcoma, glioblastoma multiforme, and CRPC, respectively^122^. Additionally, one patient with endometrial carcinoma achieved complete remission (CR) lasting for over 4 years. A more recently completed study in CRPC patients evaluated CC-115 in combination with the AR inhibitor, enzalutamide (Table 1). This combination showed no PK interactions and reduced levels of prostate-specific antigen (PSA)^123^. While positive results were seen in CRPC, CC-115 was recently discontinued in a trial in glioblastoma the Individualized Screening Trial of Innovative Glioblastoma Therapy (INSIGhT) due to concerns surrounding risk-to-benefit ratio and toxicity alongside no significant improvement in either progression-free survival or overall survival. Seven patients encountered severe and undesirable AE (i.e., Common Terminology Criteria for AE above grade 3), including abnormal liver function, cerebral edema, and hyperlipidemia (NCT02977780)^124^. While CC-115, unfortunately, failed in glioblastoma patients, furthering combination approaches in cancers that have produced favorable outcomes, such as CRPC, is necessary to evaluate this inhibitor to the greatest extent.

Some other DNA-PKcs SMIs that show promising in vitro and in vivo results have not yet undergone extensive clinical evaluation. AZD7648, a selective and potent SMI of DNA-PKcs, sensitized breast cancer and ovarian cancer patient-derived xenografts to radiation and chemotherapy^125^. Based on these findings, a phase I/IIa clinical trial evaluating AZD7648 alongside PLD was performed in patients with advanced solid tumors (NCT03907969). This trial concluded in December of 2022 and remains the only clinical trial with AZD7648 in advanced malignancies (Table 1). Lastly, XRD-0394 is an SMI with dual inhibition of ATM and DNA-PKcs developed by the private biopharmaceutical company, XRad. While no preliminary data has been released, a clinical trial using XRD-0394 for patients with metastatic, locally advanced, and recurrent tumors is underway (Table 1).

Despite the continued research and development, little progress has been made in translating DNA-PKcs SMIs into clinical settings. Unlike other PI3K proteins, such as mTOR and ATR, there are currently no U.S. Food and Drug Administration (FDA) approved DNA-PKcs inhibitors. Besides SMIs that may have off-target and toxicity issues, a novel method of DNA-PKcs inhibition is by AsiDNA, an oligonucleotide that acts as a DSB decoy within the cell. In vitro studies show AsiDNA mimicks DSBs and sequesters DNA-PKcs and other essential DDR proteins which prevents the localization of DNA-PKcs to chemo- or radiation-induced DSBs^126^. An advantage of AsiDNA over other DNA repair inhibitors is that resistance to long-term AsiDNA treatment is rarely observed^127^. Additionally, the transcriptional profile of the treated cells was altered conferring higher drug sensitivity. In a recently completed phase I trial for patients with advanced solid tumors, 2 out of 21 patients experienced dose-limiting toxicity and treatment-emergent AEs (TEAEs) were mild to moderate suggesting AsiDNA to be well-tolerated (NCT03579628)^128^. AsiDNA robustly activated DNA-PKcs and poly (adenosine diphosphate [ADP]-ribose) polymerase enzymes that induce phosphorylation of H2AX and protein PARylation. A recommended dose of 600 mg was suggested for further clinical development. AsiDNA is thus a promising new method of DNA-PKcs inhibition that is currently undergoing a phase I/II clinical trial in combination with PARP inhibitors in patients with advanced solid tumors and ovarian cancer **(**Table 1**)**.

While progress regarding DNA-PKcs’ translation into the clinic remains slow, there is still promise with SMIs, such as peposertib and CC-115. Recently, Cryo-EM structures of the human DNA-PKcs in complex with adenosine-5′-(γ-thio)-triphosphate (ATPγS) and four inhibitors, wortmannin, NU7441, AZD7648 and M3814 has been described. It was determined that the inhibitors do not structurally disturb assembly of the holoenzyme complex, instead act via direct ATP competition^129^. Hence, future drugs could be developed to target allosteric sites in addition to the conserved kinase catalytic core, to achieve better specificity for DNA-PKcs. Additionally, AsiDNA may also prove to be more effective than SMIs due to the sequestering of additional DNA repair proteins and rarely observed resistance. With the discovery of DNA-PKcs’ involvement in various cellular processes outside of DNA repair, many questions regarding its therapeutic targeting remain. How might DNA-PKcs inhibition affect cellular functioning outside of repair, such as in signaling, transcriptional, and metabolic pathways? Also, do these inhibitors have differential effects depending on the stage/grade of cancer?

### Immunotherapy and DNA-PKcs

With recent developments in ICIs, there has been a growing focus on immunotherapy. Several investigations have demonstrated that mutations in the gene encoding DNA-PKcs (*Prkdc*) are linked to an improved response to ICIs. This is primarily due to an increased tumor mutation burden (TMB) and augmented immune infiltration in tumors with *Prkdc* mutations^130^.

Analysis of previous studies related to immunotherapy found that in melanoma, NSCLC, and kidney cancer, patients with *Prkdc* mutations had response rates to ICIs of 53.8%, 75%, and 50%, respectively^131,132^. Additionally, the study employed the CT26 animal model to verify that *Prkdc* knockout cells exhibited a markedly improved response to ICIs, as evidenced by a significant reduction in tumor volume. To identify markers that may predict a better response to PD-1 inhibition, whole-genome sequencing of 34 pembrolizumab-treated NSCLC patients was performed. Out of the 14 patients who experienced a durable clinical benefit, two had *Prkdc* mutations while no similar mutations were reported in the group without a durable clinical benefit. Additionally, comprehensive correlation analysis between *Prkdc* mutations, TMB, the tumor microenvironment, and treatment outcomes in patients with various types of cancer who underwent ICI therapy showed *Prkdc* mutations were significantly linked to elevated TMB and an inflammatory tumor microenvironment.

A complicated picture is emerging from recent investigations of the cGAS/STING DNA sensing pathway in the context of cancer progression and anti-tumor immunity^133^. By priming T cell responses and increasing tumor immunogenicity, the cGAS-STING axis promotes tumor rejection^134,135^. However, STING activation also promotes metastatic dissemination^136^ and impairs the establishment of durable immunity^137^. Furthermore, although high expression of cGAS or STING predicts poor outcomes for cancer patients, downregulation of this axis has been proposed as an immune escape strategy employed by cancer cells. Similarly, while there are reports that DNA-PKcs inhibits cGAS^84^, others suggest that DNA-PKcs may be required for cGAS-STING-dependent inflammatory responses. Some of these observations are reconciled in a recent glioblastoma study, where Taffoni et al., showed that in the absence of cGAS, cytosolic DNA can activate a DNA-PKcs/IRF3-dependent type 1 interferon response^133^. However, when co-expressed, DNA-PKcs phosphorylated cGAS at S435 and synergized to optimally activate the type 1 interferon responses that promoted macrophage recruitment thus impairing early tumorigenesis. Interestingly, cGAS and DNA-PKcs levels increased with higher glioblastoma grade and correlated with poor outcomes leading the authors to speculate that long-term, sustained activation of this axis may lead to chronic inflammation which may promote tumorigenesis. Another pan-cancer study showed that inhibition of DNA-PKcs by peposertib accelerated micronucleation in irradiated p53-deficient cancer cells^138^. Cytosolic DNA thus generated, activated cGAS/STING-dependent inflammatory signaling as it elevated PD-L1 expression. When targeted with the triple combination of radiation, peposertib, and bintrafusp alfa, a fusion protein inhibiting TGFβ and immunosuppressive PD-L1 pathways, was superior to any dual combination. Hence, both the tumor microenvironment and stage/grade-dependent expression of DNA-PKcs and cGAS need to be carefully considered when devising strategies to enhance tumor immunogenicity. Nonetheless, these studies establish the DNA-PKcs complex as a potential target for combination immunotherapy as well as a predictive biomarker for immune priming of the tumor microenvironment.

## Discussion

DNA-PK, composed of the Ku70/Ku80 heterodimer and the catalytic subunit DNA-PKcs, is instrumental in safeguarding genomic stability by facilitating the repair of DNA double-strand breaks. While its classical function in NHEJ repair is well-established, recent investigations have shed light on its involvement in non-canonical signaling pathways with implications in cancer biology. By phosphorylating key proteins in the cell cycle and by evading senescence via p53 and AKT-dependent mechanisms DNA-PKc promotes cancer cell survival. DNA-PKcs promotes invasion and metastasis by regulating transcriptional networks governed by the AR and ERα respectively. By directly modulating key glycolytic enzymes and also via the mitochondrial ANT2/VDAC2 complex, DNA-PKcs supports cellular respiration. DNA-PKcs influences cancer cell metabolism and cellular energetics. DNA-PKCs activates AKT/GSK3 leading to indirect stabilization of c-MYC which supports cancer cell growth and metabolism. This expanded functional repertoire however underscores the need to consider the complexity of molecular mechanisms involved while developing targeted therapeutic strategies.

The dysregulation of DNA-PKc’s non-canonical functions has emerged as an important factor in cancer progression and in therapy resistance. Enhanced DNA-PKc activity is observed across various cancer types and correlates with aggressive phenotypes, resistance to chemotherapy, and poor patient outcomes. Despite the observable dysregulation of DNA-PKcs in cancer, the precise mechanisms underlying its contribution remain elusive, highlighting the intricacies of its molecular functions. Understanding its non-canonical roles, particularly in immune modulation, is of paramount importance, especially in the context of advancing immunotherapy approaches in oncology. Efforts to translate laboratory discoveries into clinical applications are essential. This requires a comprehensive understanding of DNA-PKcs biology and their implications. Therefore, continuous and meticulous investigation into the multifaceted roles of DNA-PKcs is crucial for developing targeted therapeutic interventions that can improve patient outcomes.

In conclusion, despite significant progress in understanding DNA-PKcs’ involvement in various biological processes, continued research is imperative to unravel its nuanced roles, particularly in cancer biology and immune modulation. This ongoing exploration is essential for unlocking the full therapeutic potential of targeting DNA-PKcs and translating these insights into actionable clinical strategies capable of positively impacting patient outcomes.

### Supplementary information

Supplementary Information
